# *Daijokito* Administration in Critically Ill Patients Increasing the Stool Volume: A Retrospective Observational Study

**DOI:** 10.3389/fnut.2021.749570

**Published:** 2021-10-11

**Authors:** Kasumi Satoh, Hajime Nakae

**Affiliations:** Advanced Emergency and Critical Care Center, Akita University Hospital, Akita, Japan

**Keywords:** Kampo, critical care, intensive care units, constipation, laxatives

## Abstract

**Introduction:** *Daijokito*, a traditional Japanese herbal medicine (Kampo), has been used to treat abdominal distention of the middle yang stage pattern. The use of *Daijokito* has not been thoroughly investigated in critical care. To investigate a new Kampo approach to defecation control in critically ill patients, our study aimed to assess the effects of *Daijokito* on fecal management.

**Methods:** We analyzed 30 consecutive patients treated with *Daijokito* in the intensive care unit (ICU) between March 2017 and February 2021. The eligibility criteria were patients who were newly prescribed *Daijokito* in the ICU during the study period. Exclusion criteria were patients who were started on other laxatives within one day of beginning *Daijokito*. The study's primary outcome was defecation volume three days before and three days after starting *Daijokito*. We recorded the most dominant stool quality within three days after the start of *Daijokito*.

**Results:** Twenty-one patients were included in the analysis. The median age was 69.0 years, and the median sequential organ failure assessment score on admission to the ICU was 6.0. Major diseases included trauma, pancreatitis, and burns. Administration of *Daijokito* resulted in defecation in 17 of twenty-one patients (81.0%). Comparison of defecation volume between 3 days before *Daijokito* administration and three days, including the day of *Daijokito* administration, showed that defecation volume increased significantly after *Daijokito* administration, with a median of 0 to 360 g (*p* < *0.001*). At the three-day follow-up, six of 17 (35.3%) patients defecated on the day of *Daijokito* administration, and nine (52.9%) defecated on the day after administration. One patient was judged to have excessive defecation, and *Daijokito* administration was discontinued. Stool quality was normal in one (5.9%) of the 17 patients, soft-formed in two (11.8%), loose-unformed in 11 (64.7%), and liquid in three (17.6%).

**Discussion:** *Daijokito* administration in critically ill patients caused defecation in 81% of the patients and significantly increased stool volume. The novelty of this study is that it sheds light on the Kampo treatment of defecation control in critically ill patients. In addition to the present report, further studies are warranted to quantify the therapeutic efficacy and safety of *Daijokito*.

## Introduction

**Figure 1:**
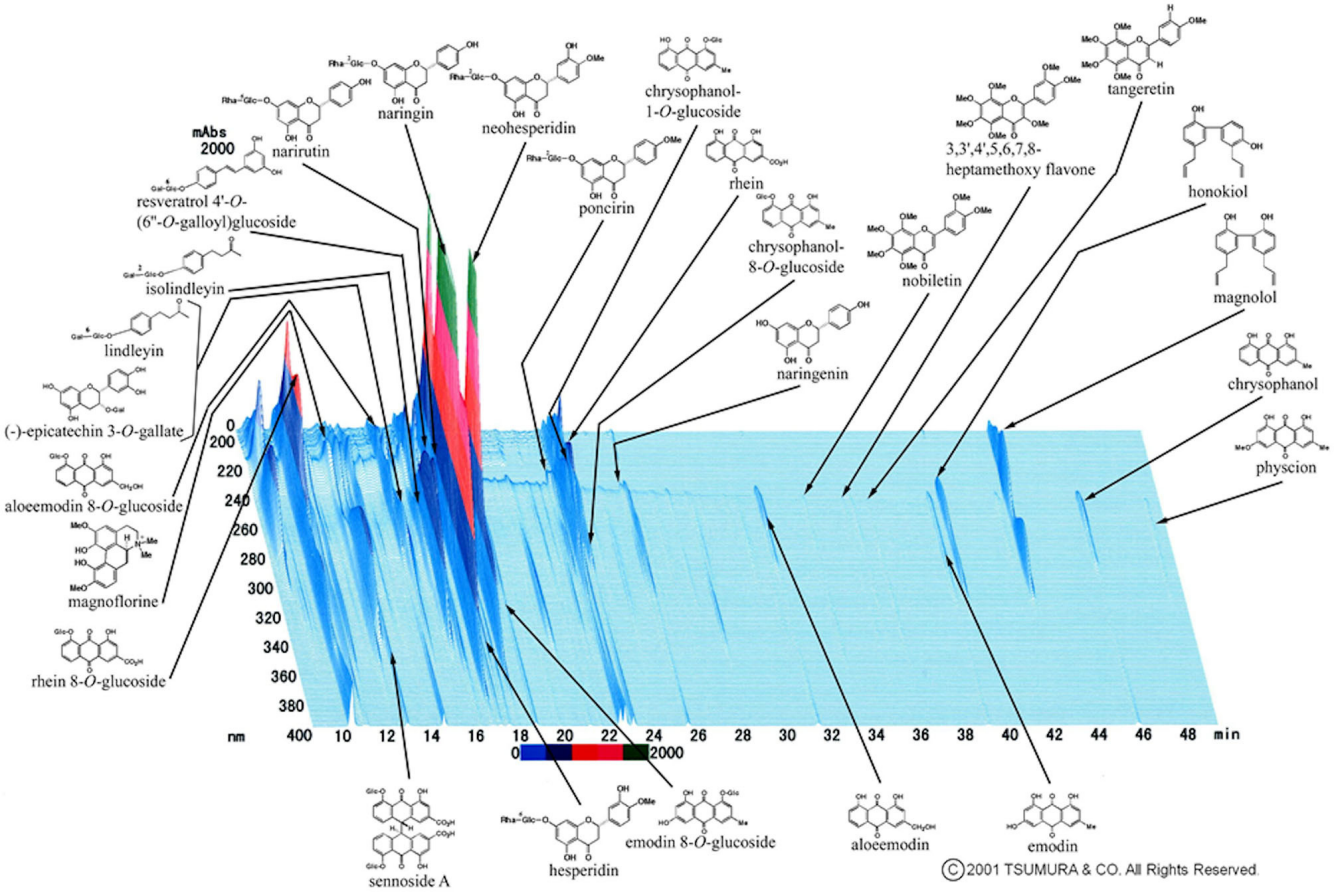
Three-dimensional high-performance liquid chromatography profile of *Daijokito* (provided by Tsumura & Co.). A three-dimensional high-performance liquid chromatography (HPLC) chart of the methanol solution of DJT. DJT was prepared with 20 mL of methanol under ultrasonication for 30 min. The solution was filtered and subjected to HPLC analysis. HPLC equipment was controlled with an HPLC pump (LC-10AD; Shimazu, Kyoto, Japan) using a TSK-GEL ODS-80TS column (4.6ϕ × 250 nm), and elution was performed using solvents (A) 0.05 M ammonium acetate (AcONH4; pH 3.6) and (B) acetonitrile (CH_3_CN). A linear gradient of 100% A and 0% B for 60 min to 0% A and 100% B was used. The flow rate was controlled with LC-10AD at 1.0 mL/min. The eluate from the column was monitored, and the 3D data were processed using a diode array detector (SPD-M10A; Shimadzu, Kyoto, Japan).

Most doctors in Japan are reported to use Kampo, traditional Japanese herbal medicine (1). The Kampo medicine *Daijokito* (DJT) is composed of the following herbs: Magnolia bark, immature orange, rhubarb rhizome, and anhydrous mirabilitum (Figure 1). In Kampo medicine, magnolia bark and immature orange regulate and normalize the flow of qi. Rhubarb rhizome and anhydrous mirabilitum have purgative properties and remove heat toxins ^[Traditionalmedicinemodule1:TM1]^ in the intestinal tract. Therefore, DJT has been used to treat abdominal distention, constipation, wheezing, and psychological symptoms in the middle yang stage pattern. Chapter 208 in Shanghanlun says that “When in middle yang stage pattern, the pulse is slow, thought there is sweating, but aversion to cold is absent, there will be generalized heaviness, shortness of breath, abdominal fullness, panting, and tidal heat effusion, which means the exterior ^[TM1]^ is about to resolve and one can attack the interior^[TM1]^. Sweat streaming from the extremities indicates that the stool is already hard, the DJT governs.” DJT is used in clinical settings to treat significant constipation, hypertension, neurosis, and food poisoning. It has also been reported as a treatment for acute pancreatitis, paralytic ileus, and tetanus in intensive care medicine (2–4).

Among Kampo medicines for gastrointestinal motility, Daikenchuto is the most investigated. Some randomized controlled trials have shown that Daikenchuto significantly enhanced ascending colon emptying compared to a placebo (5); Daikenchuto reduced postoperative ileus surgery and postoperative ileus recurrence (6). In the Japanese guidelines for nutrition support therapy in critically ill patients, Daikenchuto is expected to be a potentially effective treatment for improving gastrointestinal motility (7). However, DJT has not been well investigated. DJT contains rhubarba with stimulant laxative action (8); thus, compared with Daikenchuto, which does not include rhubarba, DJT should be a more potent agent to induce intestinal motility.

Constipation is frequently reported in 50–83% of critically ill patients (9, 10). Patients with more severe illness are more likely to have constipation (11). Constipation is reported to be associated with adverse events such as ventilator weaning failure (9) and delirium (12). Thus, defecation control in critically ill patients has important implications.

To investigate a new Kampo approach to defecation control in critically ill patients, our study aimed to assess the effect of DJT on fecal management in critically ill patients.

## Materials and Methods

### Study Design and Participants

We analyzed 30 consecutive patients treated with DJT in the intensive care unit (ICU) of Akita University Hospital between March 2017 and February 2021. Akita University Hospital is a tertiary care hospital in a rural area and has 16 ICU beds. This study was a retrospective observational cohort study and conformed to the principles of the Declaration of Helsinki. The ethics committee of Akita University Hospital approved the study protocol. The need for informed consent was admitted waiving because of the observational nature of the study and the requirement for no treatments beyond the daily clinical practice. The eligibility criteria were patients who were newly prescribed DJT in the ICU during the study period. The exclusion criteria were patients who were started on other laxatives within one day of beginning DJT.

### DJT

The decision of who to administer DJT was left to the clinician. Clinicians often initiate DJT based on the following factors: small volume of defecation, prolonged absence of stool, abdominal physiological findings, and abdominal radiographic findings. DJT was administered three times daily, one pack at a time via nasogastric tube. The DJT used in this study was produced by Tsumura & Co. (Tokyo, Japan) (8).

### Outcome

The study's primary outcome was defecation volume three days before and three days after starting DJT. In addition, the number of constipated days, number of days between the start of DJT and defecation, stool quality, and survival at discharge were recorded. If a new laxative was added before defecation, we judged that DJT was ineffective and recorded the stool volume as zero. Stool quality was classified into four categories: normal, soft-formed, loose-unformed, and liquid. We recorded the most dominant stool quality within three days after the start of DJT.

### Statistical Methods

To determine if there was a difference in each variable between the pre - and post-DJT administration, we used the Wilcoxon signed-rank test to assess the significance level at 5%. There were no missing data. Statistical analyses were conducted using Stata® software (version 16.1; StataCorp, College Station, Texas, USA). Significance was defined as a two-sided *p*-value of < 0.05.

## Results

**Figure 2:**
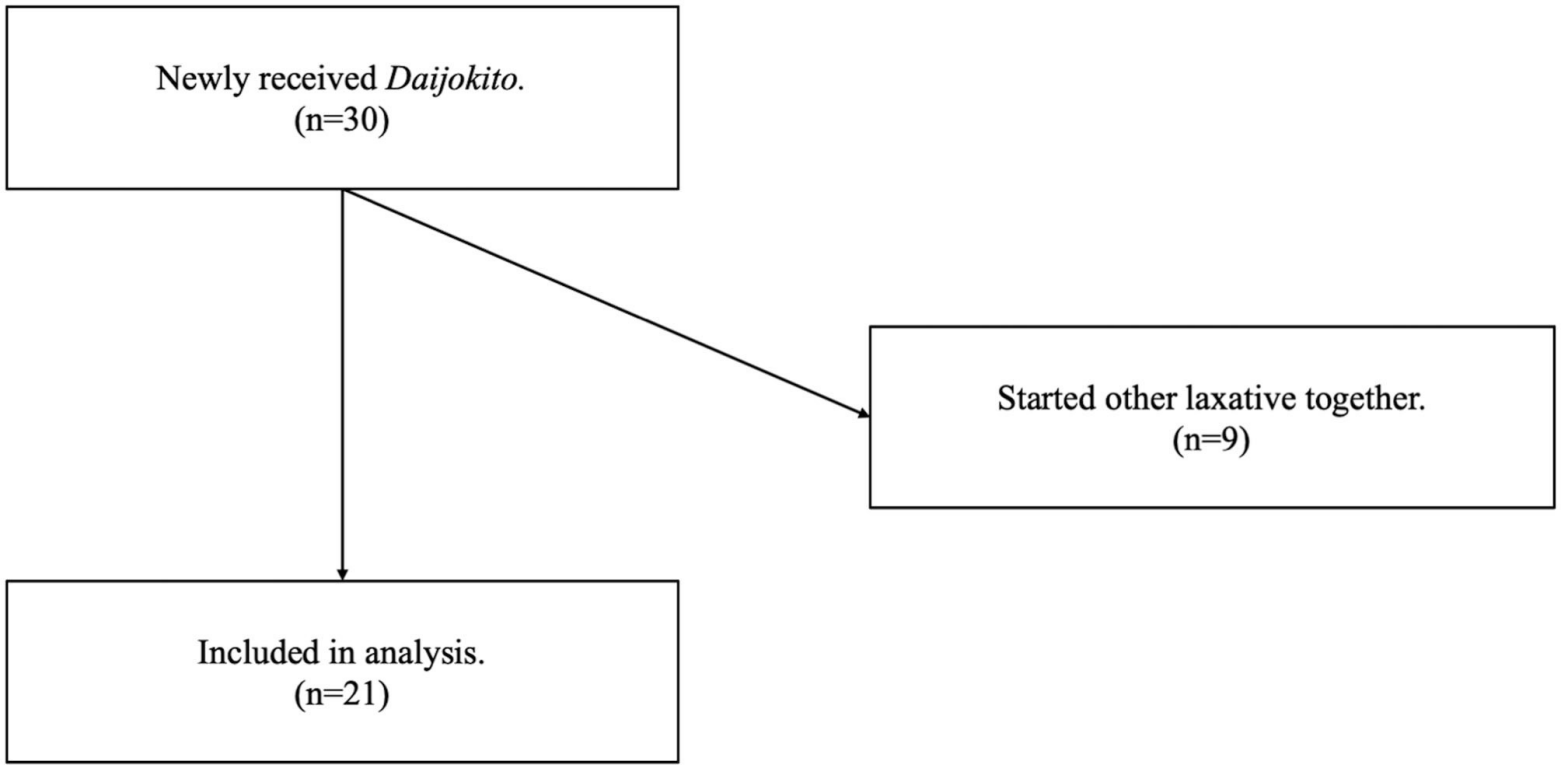
Flow of patients in a study of the effect of *Daijokito* in critical care unit.

Of the 30 patients newly received DJT in the ICU, nine were met the exclusion criteria because they started other laxative treatments at the same time. Therefore, we analyzed twenty-one patients (Figure 2). The median age was 69.0 years, and the proportion of male patients was 52.4%. The median SOFA score (13) on admission to the ICU was 6.0, and the median duration of no defecation before DJT administration was two days. The patients' major diseases included trauma, pancreatitis, burn, sepsis, tetanus, and intoxication. The patient characteristics are shown in Table 1. No patients underwent abdominal surgery.

**Table 1:** Characteristics of the patients included in the current study.

	**Variables**	
Age (years old)	69 (57–80)	
Male	11/21 (52.4%)	
SOFA score	6 (4-8)	
Constipation day (days)	2 (1-3)	
Diagnosis	Trauma	4 (19.0%)
	Pancreatitis	3 (14.3%)
	Burn	3 (14.3%)
	Sepsis	2 (9.5%)
	Tetanus	2 (9.5%)
	Intoxication	2 (9.5%)
	Heatstroke	1 (4.8%)
	Carbon dioxide narcosis	1 (4.8%)
	Acute heart failure	1 (4.8%)
	Neuroleptic Malignant Syndrome	1 (4.8%)
	Post-cardiac arrest syndrome	1 (4.8%)
Opioid use	18/21 (85.7%)	
Enteral nutrition use	20/21 (95.2%)	

*Data are expressed as medians (interquartile ranges) for continuous variables and numbers (%) for categorical variables. SOFA, Sequential Organ Failure Assessment*.

Of the twenty-one patients, we attempted to control defecation in 13 patients (61.9%) with other laxatives before DJT, five patients (23.8%) received one laxative, five patients (23.8%) received two laxatives, two patients (9.5%) received three laxatives, and one patient (4.8%) received four laxatives. As for the type of laxative, oral sodium picosulfate solution was used for six (28.6%), oral Daikenchuto, a herbal medicine, for six (28.6%), bisacodyl suppository for five (23.8%), oral naldemedine tosilate for three (14.3%), oral sennoside for two (9.5%), oral magnesium citrate solution, oral magnesium oxide, and glycerin enema for one (4.8%). As these drugs were considered inadequate, DJT was administered.

**Figure 3:**
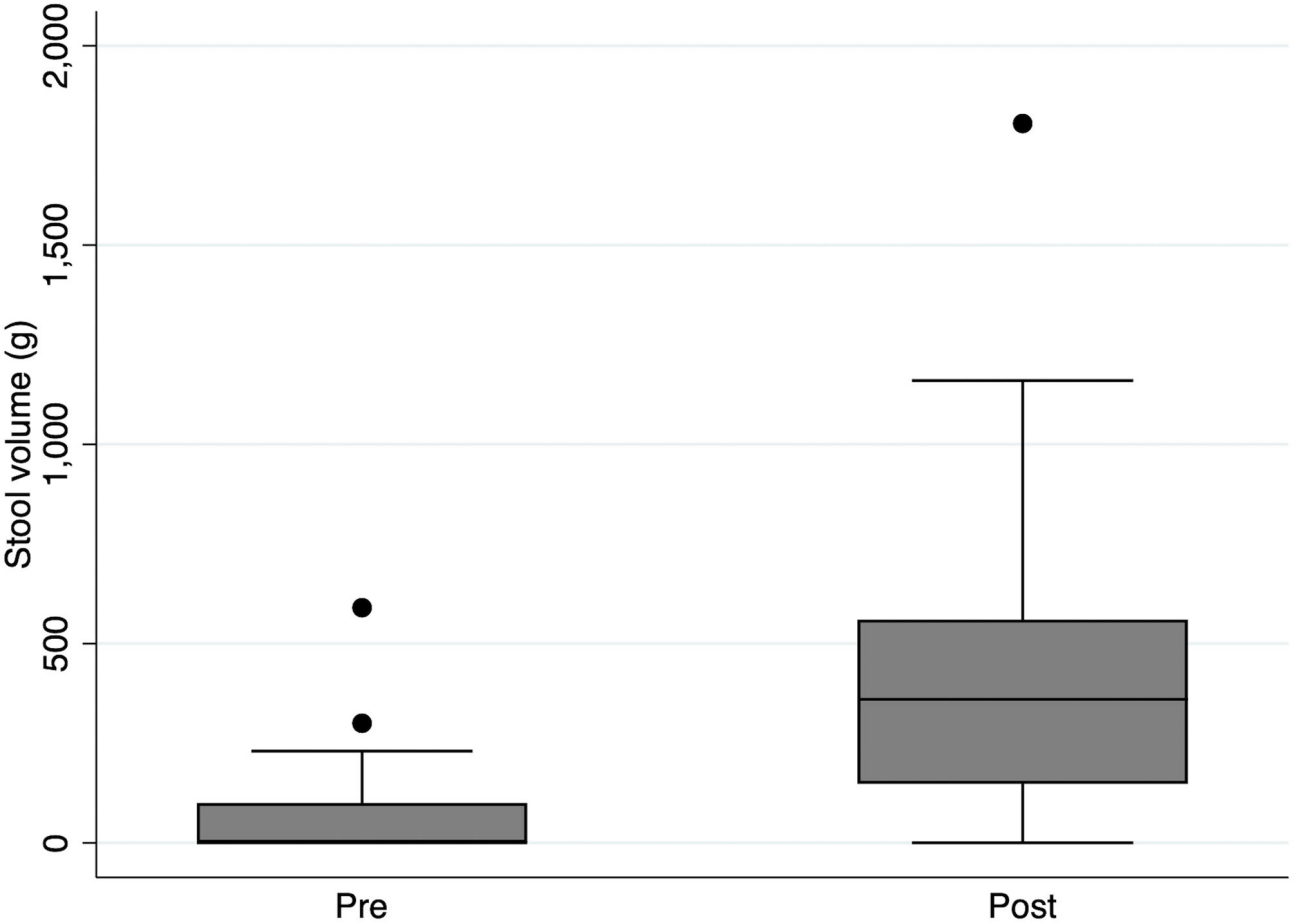
Comparison of stool volume pre and post intervention with *Daijokito*. Comparison of stool volume between 3 days before DJT administration (Pre-DJT group) and three days after, including the day of DJT administration (Post-DJT group) showed that stool volume increased significantly after DJT administration with a median of 0 g (interquartile range [IQR], 0–100) in the Pre-DJT group and 360 g (IQR, 148–560) in the Post-DJT group (*p* < 0.001).

Administration of DJT resulted in defecation in 17 of 21 patients (81.0%). Comparison of defecation volume between 3 days before DJT administration (Pre-DJT group) and three days, including the day of DJT administration (Post-DJT group) showed that defecation volume increased significantly after DJT administration with a median of 0 g (interquartile range [IQR], 0–100) in the Pre-DJT group and 360 g (IQR, 148–560) in the Post-DJT group (*p* < 0.001, Figure 3).

At the three-day follow-up, including the day of DJT administration, six of 17 (35.3%) patients defecated on the day of DJT administration, nine (52.9%) defecated on the day after administration, and two (11.8%) defecated two days after administration. One patient was judged to have excessive defecation, and DJT administration was discontinued on the second day. Stool quality was normal in one (5.9%) of the 17 patients, soft-formed in two (11.8%), loose-unformed in 11 (64.7%), and liquid in three (17.6%). There were four deaths out of twenty-one patients at discharge, with a mortality rate of 19.1%.

## Discussion

DJT administration in critically ill patients causes defecation in 81% of patients and significantly increases stool volume. Therefore, DJT is expected to improve lower gastrointestinal motility in intensive care units. Most of the defecation occurred on the day of administration or the next day, and loose-unformed was the most common stool quality. This study is novel as it is one of the first to shed light on the Kampo treatment of defecation control in critically ill patients. To the best of our knowledge, this is the first study to focus on DJT.

First, at the 3-day follow-up after DJT administration, 81% of patients showed an increase in stool volume, and more than half of these patients had bowel movement the day after DJT administration. The median stool volume increased from 0 g per 3 days to 360 g every 3 days. Few previous studies have evaluated the effect of individual laxatives on defecation volume in critically ill patients. For instance, in patients with constipation with multiple organ failure, lactulose promoted defecation in 69% of patients (median time to defecation was 36 h), and polyethylene glycol stimulated defecation in 74% of patients (median time to defecation was 44 h) (14). DJT may be competitive with these typical laxatives. Moreover, about 60% of our patients were administered DJT because other laxatives were ineffective. We propose DJT as a treatment option for refractory lower gastrointestinal motility failure in critically ill patients.

Second, loosely unformed stool was the most common stool quality, and about 82.4% of the patients presented with diarrheal stools. Almost all of our patients received parenteral nutrition. Considering that diarrhea occurred in 18% of patients receiving enteral feeding (15), the incidence of diarrhea in patients receiving DJT was high. However, only one patient (5.9%) discontinued DJT administration because of clinically determined excessive defecation, and DJT was unlikely to cause diarrhea with adverse effects, such as water and electrolyte imbalance.

The basic strategy for treating constipation is stool softening by regulating the intestinal tract's water content and enhancing bowel motility by stimulating the intestinal mucosa. The anhydrous mirabilitum in DJT has a stool softening effect, and rhubarb has a hypermotility effect, and they are traditionally utilized couplings (16). Rhubarb is reported to enhance the effect of anhydrous mirabilitum in the intestinal tract and accelerate the onset of purgative action (16). Magnolia bark has psychotropic effects (17), and immature orange has anti-inflammatory effects. Such synergistic effects and multifunctionality are the strong points of Kampo medicine. While the negative effects of polypharmacy may occur to cover various effects with western medicines, the combination of crude drugs in Kampo medicine has been sophisticated throughout history. Considering that many patients in the ICU have systemic inflammatory syndromes and that constipation is associated with delirium in critically ill patients (12), the various effects of DJT may be appropriate in the ICU.

Our study has two limitations. First, the present study was a pre- and post-observational study and did not exclude various biases and confounding factors. And the design of this study did not exclude the possibility that defecation occurred in the natural course without the use of DJT, and that the phenomenon of regression to the mean was observed. Second,the introduction/termination of DJT was left up to the clinician, which was potentially subject to bias. In particular, DJT tends to be initiated in constipation refractory to multiple laxatives, and the major limitation is the inability to distinguish the effects of various laxatives from those of DJT. In addition to the present report, further studies are warranted to quantify the therapeutic efficacy and safety of DJT.

In conclusion, the administration of DJT caused defecation in critically ill patients and significantly increased the stool volume. Although loose unformed stools were the most common stool quality, only one patient had to discontinue DJT administration. Therefore, DJT may improve lower gastrointestinal motility in patients the intensive care unit; however, further high-quality studies are needed to establish the reliability of DJT.

## Data Availability Statement

The raw data supporting the conclusions of this article will be made available by the authors, without undue reservation.

## Ethics Statement

The studies involving human participants were reviewed and approved by the Ethics Committee of Akita University Hospital. Written informed consent for participation was not required for this study in accordance with the national legislation and the institutional requirements.

## Author Contributions

KS designed the study and wrote the initial draft of the manuscript. HN contributed to the conception of the study and critically revised the manuscript. All authors contributed to the article and approved the submitted version.

## Conflict of Interest

The authors declare that the research was conducted in the absence of any commercial or financial relationships that could be construed as a potential conflict of interest.

## Publisher's Note

All claims expressed in this article are solely those of the authors and do not necessarily represent those of their affiliated organizations, or those of the publisher, the editors and the reviewers. Any product that may be evaluated in this article, or claim that may be made by its manufacturer, is not guaranteed or endorsed by the publisher.
